# Molecular factors driving the development of bovine embryos and embryo-like structures

**DOI:** 10.1590/1984-3143-AR2025-0056

**Published:** 2025-08-14

**Authors:** Zongliang Jiang

**Affiliations:** 1 Department of Animal Sciences, Institute of Food and Agricultural Sciences, University of Florida, Gainesville, FL, United States; 2 Genetics Institute, University of Florida, Gainesville, FL, United States

**Keywords:** bovine, pre-implantation development, stem cells, blastoid, blastocyst

## Abstract

Understanding the molecular mechanisms regulating the development of bovine pre-implantation embryos and formation of embryo-like structures (blastoids) is essential to uncover the causes of infertility and develop promising novel assisted reproductive technologies (ARTs). This review presents an updated view of functional genome characterization of bovine pre-implantation development. The use of genomic phenotyping and candidate gene perturbation approaches to uncover molecular factors governing bovine early embryonic development are discussed. This review also delves into the latest breakthroughs in the development of bovine blastoids and highlights key molecular signaling for the creation of bovine blastoids.

## Introduction

Early embryonic development is a very conserved process among mammals. It starts from a fertilized egg, undergoes multiple cell cleavage, and forms a pre-implantation blastocyst, where the first lineage differentiation specifies the inner cell mass (ICM) and trophectoderm (TE); the ICM further differentiates into epiblast (EPI) and hypoblast (or primitive endoderm). Subsequently, the epiblast develops into three primary germ layers, ectoderm, mesoderm and endoderm, which will form the fetus; the hypoblast or primitive endoderm gives rise to the yolk sac, which is critical to support early conceptus development; and the TE differentiates into different trophoblast cell lineages and forms the placenta. The ability of mammalian embryos to self-organize and self-differentiate into distinct yet cooperative cell lineages is a remarkable hallmark of embryonic development (Zhu and Zernicka-Goetz, 2020). However, early embryonic loss remains a major factor contributing infertility in agricultural important animals such as cattle, with significant losses occurring during the pre-implantation and peri-implantation stages (Diskin and Morris, 2008). Although the assisted reproductive technologies (ARTs) such as in vitro fertilization (IVF) have been widely used to overcome infertility or subfertility in animals, less than 50% of embryos produced by IVF develop into blastocysts in cattle, whereas in vivo derived embryos have developmental rates of 85-95% (Hasler, 2014). The reasons of inefficient largely attributes to the abnormalities of molecular programs influenced by the in vitro manipulation. Thus far, most of the molecular mechanisms during this critical period remain unknown and our understanding of how defects in this stage of development contribute to pregnancy loss remains incomplete in cattle.

Pre-implantation development is regulated by developmentally controlled events including proper changes in transcriptional, translational, and epigenetic machinery. Throughput sequencing technologies have enabled in-depth analysis of these molecular mechanisms regulating pre-implantation embryo development, including transcriptome, translatome, proteome, epitranscriptome, and epigenome (such as DNA methylation, histone modification, chromatin remodeling, non-coding RNAs). Since the publication of the bovine genome assembly (Elsik et al., 2009), ‘omics’ datasets from the bovine embryo have continued to expand in the last decade. These findings have provided molecular insights into what can potentially go wrong in pregnancies that fail at these early stages. Additionally, the characterization of key transcriptional factors regulating bovine embryogenesis and the identification of key molecular regulators that are determinants of bovine embryo competence began to immerge.

Recent years have also seen exciting progress across bovine stem cell research, including building bovine embryos from stem cell cultures, termed blastocyst-like structures, or blastoids (Pinzon-Arteaga et al., 2023; Ming et al., 2025). These advances open new frontiers for understanding of bovine early embryonic development and enabling the development of innovative ARTs aimed at enhancing bovine reproductive efficiency.

In this review, first, the molecular factors driving the bovine pre-implantataion development is extensively discussed, including an updated view of functional genome characterization and the use of genomic phenotyping and candidate gene perturbation approaches to uncover molecular drivers. Second, the recent advances in the derivation of bovine stem cell-based embryos (blastoids) are summarized. Particularly, the key molecular signaling for the creation of bovine blastoids and the proposed key steps to improve blastoid development are highlighted.

## Molecular factors driving the development of bovine pre-implantation embryos

Upon fertilization of a matured egg, bovine embryos undergo a series of cleavage divisions. The maternal stored RNAs/proteins degrade, and embryonic genome becomes activated by the 8-cell stage in bovine embryos. It is followed by compaction and polarization, which occurs between 16- to 32-cell stage, and subsequently formation of a cavity, the blastocoel, which denotes the formation of the blastocyst. It is believed that the general molecular mechanisms driving mammalian pre-implantation embryo development are conserved and much what we have learned from model systems such as mice can be inferred in bovine. However, species specific mechanisms exist and understanding the unique molecule features of bovine pre-implantation development is fundamental to trickling the remarkable problems of embryonic loss and early pregnancy failure in the cattle reproductive field.

## Updated landscapes of functional genome with increasing resolution

**Figure 1:**
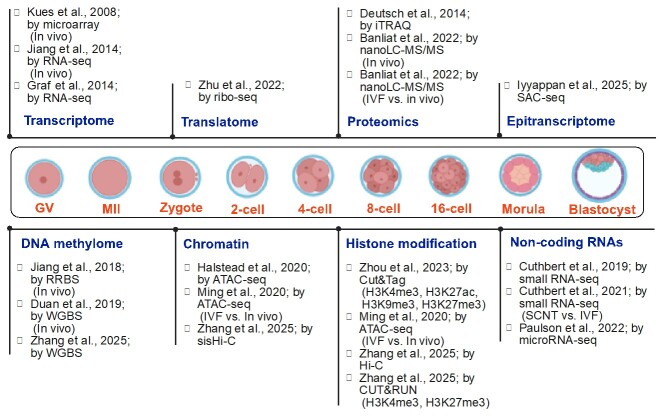
Reports of functional genome characterizations in bovine oocytes and different stages of pre-implantation embryos.

Throughput sequencing technologies enables the characterization of functional genome of pre-implantation embryo development at unprecedented level. These include transcriptome, translatome, proteome, and epitranscriptome, as well as epigenome (such as DNA methylation, histone modification, chromatin remodeling, non-coding RNAs) (Figure 1).

Characterization of whole transcriptome was among the first step to establish precise gene transcription programs during bovine pre-implantation development. It was first reported by using the microarray technology but limited gene detection to only those printed on the array (Kues et al., 2008). Thereafter, high-throughput RNA sequencing (RNA-seq) has provided comprehensive profiling of all transcripts expressed during bovine pre-implantation development (Graf et al., 2014; Jiang et al., 2014). These studies have revealed that the timing of major embryonic activation (EGA) in bovine occurs between the 8- and 16-cell stages in embryos derived in vitro, while at 4- to 8-cell transition in embryos derived in vivo. They have also identified differentially expressed genes during the consecutive stages of each developmental transition, a vast amount of little-known potential regulators during bovine pre-implantation development.

However, the mRNA abundance does not necessarily represent its functional status (protein abundance) (Becker et al., 2018). Therefore, proteomic studies are indispensable to understand the molecular mechanisms governing early embryonic development. The characterization of proteomics of bovine pre-implantation development has been explored (Deutsch et al., 2014; Banliat et al., 2022a, b). Particularly, using a nano liquid chromatography coupled with label free quantitative mass spectrometry, the dynamics changes of proteins across bovine pre-implantation development has been analyzed with embryos produced in vivo (Banliat et al., 2022a, b). This analysis adds a valuable dataset for further data mining and infers biochemical functional characterizations of molecular regulators during bovine embryo development.

Translational control regulates protein synthesis by modulating mRNA translation initiation, elongation, and termination (Gebauer and Hentze, 2004). Therefore, the understanding of mRNA translational dynamics is also pivotal during pre-implantation development. This gap has been filled with a comprehensive analysis of translational landscapes of bovine pre-implantation embryo development using a high-resolution ribosome profiling approach (Zhu et al., 2022). In this study, non-translated RNA (free mRNA), mRNA prepared for translation (different numbers of monosomes-bound mRNA), and actively translating mRNA (different numbers of polysomes-bound mRNA) of bovine pre-implantation embryos have been systematic characterized. It reveals four modes of translational selectivity: (1) selective translation of non-abundant mRNAs; (2) active, but modest translation of a selection of highly expressed mRNAs; (3) translationally suppressed abundant to moderately abundant mRNAs; and (4) mRNAs associated specifically with monosomes. This resource allows to dissect the dynamic of specific mRNA that is untranslated, translated, or degraded and the translation selectivity of individual mRNA during bovine early embryogenesis, therefore adding a missing picture of translational regulation of bovine pre-implantation development.

Many forms of modifications exist in RNA and play key regulatory roles in gene transcription. For example, N^6^-methyladenosine (m^6^A) is the most abundant internal modification on transcribed mRNAs. Emerging evidence have suggested that m^6^A plays a crucial role in early embryonic development by regulating RNA stability and degradation, particularly during the maternal-to-zygotic transition (Zhao et al., 2017). In a recent study, by using a newly developed m^6^A-selective allyl chemical labeling and sequencing (m^6^A-SAC-seq) approach (Hu et al., 2022), the transcriptome-wide landscapes of m^6^A in bovine oocytes (GV and MII stages), and pre-implantation embryos (4-cell, 8-cell, 16-cell, and blastocyst stages) at single base resolution have been described (Iyyappan et al., 2025 forthcoming). It is found that the m^6^A abundance is peaked at GV oocytes but exhibits a marked drop during oocyte maturation and across cleavage stage embryos, and finally gradually increases at blastocysts. It is also reported that m^6^A marks maternal transcripts largely reflecting the suppression of transcription and translation in oocytes, then this modification is lifted in embryos at 4-, 8-, and 16-cells to boost the activation of embryonic genome, and finally selectively marks genes for earlier lineage differentiation. Genes with abundant m^6^A modification that specifically associated with each developmental stages have also been identified, indicating their key regulatory roles.

The epigenetic mechanisms also regulate gene expression. The rapid emergence of epigenomic analysis of bovine embryos generates significant datasets, enabling our understanding of critical epigenetic reprograming events during bovine pre-implantation development. First, DNA methylation is an epigenetic mechanism and serves to regulate gene transcription for embryonic development, gene imprinting and X-chromsome inactivation (Li et al., 1993; Bird, 2002; Jaenisch and Bird, 2003). DNA 5mC methylation dynamics have been characterized in bovine embryos by using reduced representative bisulfite sequencing (RRBS) and whole genome bisulfite sequencing (WGBS). Both analysis of bovine in vivo derived pre-implantation embryos (Jiang et al., 2018; Duan et al., 2019) have concluded that 1) the major wave of genome-wide DNA methylation is completed by the 8-cell stage, 2) the sperm and oocytes are differentially methylated in numerous regions (DMRs), and 3) DMRs are identified between in vivo and in vitro matured oocytes, providing the regions that are sensitive to the environmental stress. An informative dataset of DNA methylome of bovine oocytes and pre-implantation embryos derived in vitro has also generated recently (Zhang et al., 2025).

Second, both chromatin accessibility and high-order structure organization play important roles in gene expression regulation. The dynamics of accessible chromatin has been defined in bovine embryos using ATAC-seq (an assay for transposase-accessible chromatin) (Halstead et al., 2020; Ming et al., 2021). Both studies suggest that bovine embryos experience progressive chromatin accessibility during cleavage, which is consistent with the degradation of the maternal transcripts and activation of the embryonic genome. Additionally, they have also identified the TFs that are important to drive bovine pre-implantation development. Most recently, 3D chromatin architecture of bovine oocytes and pre-implantation embryos has also been characterized by using a Hi-C technique (Zhang et al., 2025). It is shown that 3D chromatin architecture is dispersed and gradually reestablished during bovine pre-implantation development (Zhang et al., 2025).

Third, histone modifications are essential for regulating spatiotemporal gene expression in early development and different types of histone modifications have diverse functions (Suganuma and Workman, 2011). For example, H3K4me3 and H3K27ac are active marks for promoters and transcription, while H3K9me3 and H3K27me3 are repressive histone marks. The locus specific localization of histone modifications (two activating: H3K4me3 and H3K27ac and two repressive: H3K9me3 and H3K27me3) marks across bovine oocytes and different stages of pre-implantation embryos has been described using a CUT & Tag (Zhou et al., 2023) and Cut & Run (Zhang et al., 2025). It is found that broad bivalent domains mark developmental genes in bovine oocytes, H3K9me3 and H3K27me3 co-occupy gene bodies prior to EGA, while chromatin accessibility is established before canonical H3K4me3 and H3K27ac signatures during EGA. H3K27me3 is also found to play a major role in restriction of cellular potency at blastocyst stage.

Forth, non-coding RNAs (ncRNAs) are emerging as key regulators of embryogenesis by regulating embryonic gene expression (Pauli et al., 2011). Comprehensive profiling of ncRNAs has been conducted in different stage of bovine pre-implantation embryos (Cuthbert et al., 2019, 2021; Paulson et al., 2022). It is found that miRNA abundance is elevated at the 8-cell stage when the major EGA occurs, while piRNAs are found in bovine oocytes and blastocysts, but not in 8-cell stage embryos. Interestingly, there is a shift of sncRNA abundance at the 8-cell stage (Cuthbert et al., 2019) and the embryonic miRNAs are dramatically increased their expression during the morula and blastocyst stages (Paulson et al., 2022).

## Molecular and genomic phenotyping

In addition to the comprehensive characterization of functional genome, analyzing embryo molecular phenotypes resulting from various stresses or treatment conditions at different developmental stages is critical for the identification of the key drivers control early embryonic development. By comparative analysis of bovine embryos derived in vivo, in vitro, and somatic cell nuclear transfer (SCNT), a number of molecular candidates have been identified with respects of their mRNA expression, protein abundance, DNA methylation, histone modification, chromatin accessibility, and small ncRNAs (reviewed in Zhu et al., 2021; Jiang 2023). For example, in a recent single cell transcriptomic comparative analysis of bovine blastocysts derived in vivo (IVV), in vitro from a conventional culture medium (IVC), and in vitro from an optimized reduced nutrient culture medium (IVR) (Ming et al., 2024), it was found that bovine IVC embryos process highly active metabolic and biosynthetic processes, reduced cellular signaling, and reduced transmembrane transport activities, while IVR embryos have lower activities in metabolic and biosynthetic processes but increased cellular signaling and transmembrane transport. Additionally, the IVR embryos had compromised development compared to IVV embryos with notably over-active transmembrane transport activities that impaired ion homeostasis. This study comprehensively determines how in vitro culture conditions impact bovine blastocyst quality at the transcriptomic level. Therefore, the ability to utilize transcriptomic, translatomic, epigenomic, and proteomic approaches to generate comprehensive datasets would allow the identification of the key regulators controlling bovine pre-implantation development, and pave the way for future optimization of bovine embryo technologies.

## The candidate gene approach

The identification of key molecular factors driving bovine pre-implantation development is critical for developing promising strategies to improve embryo competence and pregnancy success. While regulators arrayed from genetically modified mouse models have been valuable, uncovering relevant mechanisms for bovine pre-implantation development remains challenging due to fundamental physiological differences among different species. Thus far, a number of genes have been functional characterized during bovine embryogenesis, including lineage specification transcriptional factors, methyltransferases/demethylases, microRNAs, as well as other genes and signaling act at various developmental stages (reviewed in Zhu et al., 2021; Jiang, 2023). Most of the studied genes, upon perturbation, halt bovine pre-implantation development. However, beneficial molecules have also been reported. By studying the role of a nuclear coding gene, methyltransferase-like protein 7A (METTL7A) in bovine early development, it is found that METTL7A improves the developmental potential of bovine embryos (Zhu et al., 2025). Mechanistically, the exogenous METTL7A modulates expression of genes involved in embryonic cell mitochondrial pathways and promotes trophectoderm development. METTL7A alleviates mitochondrial stress and DNA damage and promotes cell cycle progression during embryo cleavage. In summary, this study has identified a novel mitochondria stress eliminating mechanism regulated by METTL7A that occurs during the acquisition of oxidative stress in embryo in vitro culture. Upon further development of the gene delivery approach, this discovery lays the groundwork for the development of METTL7A as a promising molecule target for improving bovine IVF embryo competence.

Understanding the dynamics of functional genome during bovine pre-implantation development continues to immerge, mostly due to the ever-evolving high resolution omics technologies. Further understanding the complex gene regulation and the interplay and cooperative role of each level of omics to drive bovine pre-implantation development are essential. With the recent advancements in genome editing technologies, such as CRISPR/Cas9 system, we now can rapidly introduce targeted genetic modifications and characterize the role of individual candidates during bovine pre-implantation development. Only after we understand the role of each of these regulators in bovine early development at the same level and scale as they have performed in mice models, we can begin to explore the intervention strategy and take advantage of the positive, adaptive aspects of developmental programming to improve bovine embryo competence. Additionally, machine learning has been utilized to identify the biomarkers of bovine embryo competence (Rabaglino et al., 2023), offering a powerful platform for the development of novel biomarkers of embryo competence.

## Molecular signaling driving the development of bovine blastoids

After a series of cleavage divisions, a single blastomere from a zygote stage embryo can generate a blastocyst composed of three founding tissues - epiblast, hypoblast, and trophoblast - to form the body plan. Surprisingly, recent research has shown that the embryo-derived stem cells, when cultured in confining microwells under specific culture conditions, retain a similar generative capacity to self-organize into blastocyst-like structures, or blastoids, as first reported in mice (Rivron et al., 2018; Li et al., 2019; Sozen et al., 2019). Blastoids consist of all the founding cell lineages of the fetus and its supporting tissues, similar to blastocysts, which hold significant promise for advancing our understanding and enhancement of animal reproduction. In cattle, with the decade-long efforts, researchers have successfully derived different types of embryo-derived stem cells including embryonic stem cells (ESCs) (Bogliotti et al., 2018), expanded pluripotent stem cells (EPSCs) (Zhao et al., 2021), trophoblast stem cells (TSCs) (Wang et al., 2023), and extraembryonic endoderm stem cells (XENs) (Ming et al., 2025), as well as induced pluripotent stem cells (iPSCs) reprogrammed from somatic cells (reviewed in (Su et al., 2020; Weeratunga et al., 2023). These stem cell lines not only provide *in vitro* models to enhance our understanding of bovine early embryonic development but also serve as crucial building blocks for developing bovine stem cell-based embryo models, e.g., blastoids.

**Figure 2:**
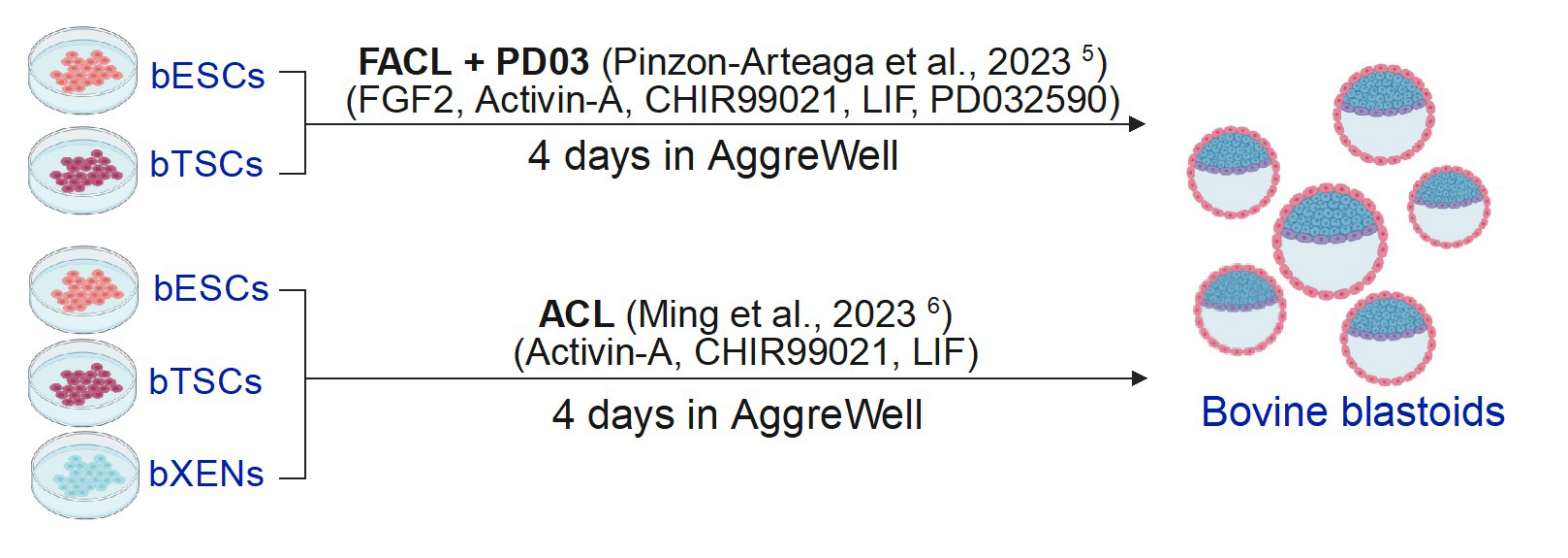
Two published protocols to generate bovine blastoids.

Thus far, there are two different protocols developed to create self-organizing blastoids in cattle, either by using a robust medium termed tFACL + PD: (FGF2, activin A, CHIR99021, LIF, PD0325901) to assemble bovine EPSCs and TSCs within four days – bovine EPT-blastoids (Pinzon-Arteaga et al., 2023), or by using ACL (Activin-A, Chir99021, LIF) to assemble bovine EPSCs, TSCs and XENs – bovine EPTX-blastoids (Ming et al., 2025) (Figure 2). It has been shown that EPT-blastoids resemble blastocysts in morphology, cell composition, single-cell transcriptomes, *in vitro* growth, and the ability to elicit maternal recognition of pregnancy following transfer to recipient cows (Pinzon-Arteaga et al., 2023). However, several differences have been observed between bovine EPT-blastoids and blastocysts, e.g., proportions of EPI and HYPO lineages – EPT-blastoids have a lower proportion of hypoblast-like cells and a higher number of epiblast-like cells compared to the IVF blastocyst (Pinzon-Arteaga et al., 2023). By including bovine XENs, it has been demonstrated that bovine EPTX-blastoids more closely resemble bovine blastocysts compared to EPT-blastoids in terms of morphology, lineage composition and allocation, and transcriptional features (Ming et al., 2025).

Besides of the establishment of the building blocks – EPSCs, TSCs, and XENs, the process to induce bovine blastoids essentially relies on manipulating the external signal pathways to optimal three cell lineage components of blastoids – epiblast-, trophoblast- and hypoblast-like cells. In the bovine EPT-blastoids induction, researchers first adapted a FAC medium (FGF2, Activin-A and CHIR99021) (Yu et al., 2021a) that supports the differentiation of hypoblast-like cells from naïve human PSCs (Yu et al., 2021b), and added the LIF that is known to improve bovine pre-implantation embryo development (Stoecklein et al., 2021). As the FGF signaling level can bias the ICM through the MEK-ERK pathway (Turner and Grose, 2010; Lavoie et al., 2020), where high level of FGF directs ICM cells towards the hypoblast lineages (Rossant and Tam, 2017). To support both hypoblast and epiblast lineages, additional FGF signaling optimization has been conducted by lowering FGF2 concentration and including a low dose of a MEK inhibitor (PD0325901, 0.3µM), as MEK inhibition has been shown to suppress hypoblast fate in bovine embryos in a dose dependent manner (Canizo et al., 2019). The final optimized condition termed titrated FACL+PD03 (tFACL+PD) enabled the efficient and robust EPT-blastoid formation within 4 days. The optimization the culture condition for creating bovine EPTX-blastoid is essentially based on the tFACL+PD condition. It is reported that although the blastoid formation is very efficient under this condition, they have vanished hypoblast lineages compared to IVF blastocysts, similar as seen in EPT-blastoids (Ming et al., 2025). Given that FGF2 could bias the cell fate of ICM towards PrE (Kuijk et al., 2012; Rossant and Tam, 2017; Pinzon-Arteaga et al., 2023), and the FGF2 is not necessary due to the integration of XEN cells, as well as MEK inhibitor PD0325901 inhibits hypoblast specification from ICM (Kuijk et al., 2012; Canizo et al., 2019), the researchers withdrawn both FGF2 and PD0325901 from the tFACL+PD condition, and they concluded that the modified medium, ACL (Activin-A, Chir99021, LIF) enables the efficient and robust formation of blastoids morphologically more resemble day 8 IVF blastocysts compared to the EPT-blastoids (Ming et al., 2025).

Thus far, it is still unclear whether and how bovine blastoids can be generated using bovine ESCs as the solely starting cells as described in other species. This is largely due to the lack of bovine naïve ESCs, that has seen in mice (Evans and Kaufman, 1981; Martin, 1981) and rats (Buehr et al., 2008; Li et al., 2008) with unfettered developmental capacity to contribute into all somatic and germline lineages. Notably, a recent study systematically investigated the roles of the LIF/STAT3, WNT, AND FGF/MARK pathways and developed a universal serum-free 6iL culture system that enables the derivation and long-term self-renewal of ESCs across five mammalian species including cattle (Wang et al., 2025). Inducible expression of KLF2 and NANOG further enhances the naïve pluripotency and chimeric potential of bovine ESCs (Wang et al., 2025). Given that the quality and efficiency of blastoid formation are heavily influenced by the characteristics of the starting cell types, it is anticipated that this naïve like bovine ESCs can be self-organized into blastoids with increased developmental potential.

Future investigations to achieve better blastoids and the application of blastoid in cattle reproduction are warranted. To advance the field, first, it is critical to invest in uncovering the unique signaling pathways and molecular programs that guide lineage segregation, specification, and differentiation during bovine embryogenesis. Second, current methods for the creation of bovine blastoids rely on the biochemical optimization of media with lineage-specific morphogens or small molecules (Pinzon-Arteaga et al., 2023; Ming et al., 2025). In the model species like mice, however, it has been shown that stem cell embryo models can also be created by a combination of exogenous transcription factors-induced cells and natural embryo-derived cell lines (Langkabel et al., 2021; Tarazi et al., 2022; Dupont et al., 2023), or through intrinsic epigenetic control – CRSPR activation of two regulatory elements near GATA6 and CDX2, and without dependency on externally added lineage-specific signaling factors (Lodewijk et al., 2025). It is anticipated that such approaches can be utilized to create bovine blastoids. Third, it is important to comprehensively analyze molecular programs (e.g., functional genome) of bovine blastoids, compared to blastocysts. Such information is essential for optimizing blastoid induction conditions and unlocking the full potential of bovine blastoid technology in both research and reproduction. Finally, achieving successful pregnancies or even live births from bovine blastoids, if possible, will require years of efforts to come.

## Concluding remarks

A marvelous array of powerful technologies becomes available to investigate the development of pre-implantation embryos at the molecular and cellular levels. Although this line of research is largely delayed in domestic livestock such as cattle, it catches up rapidly. The identification of key transcriptional factors of bovine embryogenesis and ultimately exploring the intervention strategies to improve bovine embryo survival are desperately needed. These molecular strategies combined with synthetic biology will help us to gain a comprehensive network of molecular factors driving the bovine early embryonic development. Bovine blastoids, while are artificially maintained and still in their infancy, offer an invaluable resource for studying bovine embryonic development. Upon further development, this technology also holds great potential to transform the fields of bovine embryo technologies.

## Data Availability

Data sharing is not applicable as no new data were generated or analyzed during this study.
